# Deciphering Chemical Rules for Drug Penetration into Strongyloides

**DOI:** 10.3390/pharmaceutics16091224

**Published:** 2024-09-19

**Authors:** Miguel Marín, Javier Sánchez-Montejo, Sergio Ramos, Antonio Muro, Julio López-Abán, Rafael Peláez

**Affiliations:** 1 Laboratorio de Química Orgánica y Farmacéutica, Departamento de Ciencias Farmacéuticas, Universidad de Salamanca, Campus Miguel de Unamuno, E-37007 Salamanca, Spain; mmarin@usal.es (M.M.); sergio.rvarela@usal.es (S.R.); 2 Instituto de Investigación Biomédica de Salamanca (IBSAL), Facultad de Farmacia, Universidad de Salamanca, Campus Miguel de Unamuno, E-37007 Salamanca, Spain; 3 Centro de Investigación de Enfermedades Tropicales de la Universidad de Salamanca (CIETUS), Facultad de Farmacia, Universidad de Salamanca, Campus Miguel de Unamuno, E-37007 Salamanca, Spain; 4 Infectious and Tropical Diseases Research Group (e-INTRO), Biomedical Research Institute of Salamanca Research Centre for Tropical Diseases at the University of Salamanca (IBSAL-CIETUS), E-37007 Salamanca, Spain; s.montejo@usal.es (J.S.-M.); ama@usal.es (A.M.)

**Keywords:** *Strongyloides venezuelensis*, dyes, nematocidal activity, structure–activity relationships, entry rules

## Abstract

**Background:** Strongyloidiasis, a parasitic infection, presents a significant public health challenge in tropical regions due to the limited repertoire of effective treatments. The screening of chemical libraries against the therapeutically relevant third-stage larvae (L3) of the model parasite *Strongyloides venezuelensis* yielded meager success rates. This situation is reminiscent of Gram-negative bacteria, where drug entry is a limiting factor. **Methods:** Here, we set out to determine whether similar barriers are in place and establish whether structural and property requirements exist for anti-strongyloides drug discovery. We focused on dyes as their uptake and effects on viability can be independently assessed in the multicellular parasite, thus providing a means to study the possibility of similar entry rules. We tested different dyes in in vitro assays on L3s. **Results:** We found that staining was necessary to reduce parasite viability, with some dyes achieving anti-strongyloides effects at concentrations similar to those of the reference drug, ivermectin (IV). Some dyes also showed activity against female adults at concentrations well below that of ivermectin. Unfortunately, the most potent dye, Methylene Blue, was unable to prevent the infection in a preliminary in vivo mouse model assay, presumably due to fast dye clearance. Structural analysis showed that positive charges facilitated the access of the compounds to the L3 tissue, thus providing a structural tool for the introduction of activity. For female adults, low globularity is additionally required. As a proof of concept, we added a positive charge to an inactive compound of one of our chemical libraries and re-determined the activity. **Conclusions:** These findings allow us to establish structural rules for parasite entry that could be of interest for future drug screening or drug development campaigns. These rules might also be applicable to other related parasites.

## 1. Introduction

Infections caused by soil-transmitted helminths (STH) are a major global health issue, being especially prevalent in developing regions. The World Health Organization (WHO) estimates that 20% of the global population has possibly been affected by at least one of them [1,2], including *Ascaris lumbricoides*, *Trichuris trichiura*, hookworms, and *Strongyloides stercoralis*. Strongyloidiasis, caused by *Strongyloides* species, is a persistent and silent infection, affecting nearly 600 million people, mainly in tropical areas; it causes gastrointestinal, cutaneous, and pulmonary symptoms, as well as retarded growth in children [3]. *Strongyloides stercoralis* has a complex life cycle, encompassing free-living and parasitic stages. In mammalian hosts, third-stage larvae (L3) penetrate through the skin, migrate to the lung, and then reach the small intestine, where they mature into parasitic female adults. These parthenogenetic females burrow into the gut wall and lay eggs that hatch in the mucosa, resulting in first-stage larvae that contaminate the environment through feces. Some of these larvae do not leave the host but migrate through the body, causing autoinfection. Adult females and larvae coexist in the host, thus constituting the therapeutically relevant lifecycle stages [4]. *Strongyloides stercoralis* can also cause a systemic disseminated infection or hyperinfection syndrome (HS) in immunocompromised patients, where the available treatments become inefficient [5,6].

Strongyloidiasis treatment in humans relies mainly on only three drugs [7]: the macrocyclic lactones ivermectin (IV) [8] and the benzimidazoles albendazole and mebendazole [9,10]. In the last few decades, only a few drugs, such as emodepside, monepantel, and derquantel, have received approval to target parasitic nematodes, but only for veterinary purposes [11]. Moreover, the systematic use of these drugs to control gastrointestinal nematodes in grazing farm animals has led to a surge in resistance to benzimidazoles and macrocyclic lactones throughout the world [12,13]. Future programs to control human gastrointestinal infections by nematodes should consider these limitations.

In the context of the reduced number of nematicides, new treatments are needed to control human gastrointestinal infections by nematodes, but challenges of a different nature impose severe limitations [14]. The development of new drugs for parasitic diseases is plagued by scientific challenges related to the drugs’ absorption, distribution in the organism, entry into the nematodes, and eventual killing of the parasite. A screening campaign of structurally diverse drugs in our research group against L3 of *Strongyloides venezuelensis*, a validated model for *S. stercoralis*, yielded very poor success rates, much lower than similar assays on other parasites, such as *Leishmania* spp. [15], *Trichinella spirallis*, or *Schistosoma* spp. This unusually low hit rate resembles the limited success achieved with drug screens against Gram-negative bacteria [16], although much less effort has been dedicated to drug discovery for strongyloidiasis. The gap in drug discovery against Gram-negative bacteria is linked to the inability of most drug leaders to accumulate inside due to the combined barriers of their two cellular membranes, especially the lipopolysaccharide-coated outer one, which require active small molecules to cross through the narrow polar channels of the porins and avoid the efflux pumps [16]. These uptake barriers severely restrict the properties of active compounds, thus shaping the chemical space for drug penetration and allowing the establishment of chemical rules for good drug accumulation and activity [17]. These rules allow the transformation of inactive compounds into active ones by merely modifying their properties and therefore their uptake [18]. We reason that similar barriers could be in place for the *Strongyloides* L3 or parthenogenetic females, where the cuticle could combine with the membrane to similarly restrict the chemical space for drug penetration into the nematode, as previously suggested [14]. The importance of drug uptake and permeability for drug action is well established, but drug accumulation studies are more difficult to perform in multicellular parasites than in bacteria or other unicellular organisms [19,20]. Cell staining with dyes is a classical means to determine uptake, and the methodology is extensible to multicellular parasites, where differential staining is often of histological significance. Furthermore, dyes can have different effects on cells: some dyes are non-toxic, whereas others can have toxic effects on the cells, thus decoupling both effects—uptake and toxicity.

Dyes find use mainly for diagnosis and medical imaging, but many of them have been also used as drugs. Methylene Blue was the first synthetic drug used in medicine and is still used today for the treatment of methemoglobinemia (ProvayBlue^®^) and has been proposed as a possible drug for Alzheimer’s disease [21]. Moreover, this dye is used for the antibacterial photodynamic inactivation of nosocomial infections [22,23] and the treatment of sepsis of the urinary tract (Urelle^®^) [24]. Moreover, it was used against protozoa such as *Neospora caninum* [25] and for a long period of time as an antimalarial [26]. Other dyes, such as Crystal Violet, have also shown interesting antimicrobial and anti-parasitic properties [27]. The possibility of tracking the molecule inside the body is another interesting quality of dyes as drugs, especially in the case of parasitic diseases, because it allows the monitoring of the ways in which they are absorbed, distributed, metabolized, and excreted, not only in the host but also in the parasite.

In this study, we investigated the uptake and efficacy of a wide range of dyes against third-stage larvae (L3) and parasitic adults of *S. venezuelensis*, a model for *Strongyloides stercoralis*, and in experimental infections. There is a relationship between dye uptake and activity, which are sequential. We analyzed the structural characteristics necessary for uptake and activity and found that cationic compounds are taken up and are able to kill the parasites, whereas neutral or anionic ones fail to do so. For adult females, a positive charge was not sufficient and low globularity was additionally required for uptake (assessed by staining) and activity (assessed by shape and motility). This allowed us to establish chemical rules for entry into the parasite. As a proof of concept, we selected a low-globularity compound that failed to kill the L3 larvae in our library screens but succeeded against other parasites, and we modified it to conform to the rules. The positively charged analog killed the L3 larvae and affected the adults. These results show that we can establish entry rules for *Strongyloides* similarly to Gram-negative bacteria. The rules will help to configure chemical libraries for drug screening and convert inactive compounds into active ones, thus increasing the success rates in the search for new drugs against this parasite.

## 2. Materials and Methods

### 2.1. Drugs and Sample Preparation

Methylene Blue, Crystal Violet, Fuchsine, Azur–Eosin–Methylene Blue, Carmine, Sudan III, Congo Red, Eriochrome Black T, Methyl Orange, and Hematoxylin were purchased from ITW Reagents Panreac (Barcelona, Spain); Brilliant Cresyl Blue was purchased from Analema (Pontevedra, Spain); Indigo Carmine from Geigy AG (Basel, Switzerland); and Trypan Blue, propidium iodide, and fluorescein diacetate from Sigma (St Louis, MO, USA). Ivermectin in powder form and albendazole and mebendazole were from Sigma and edelfosine was from R. Berchtold (Biochemisches Labor, Bern, Switzerland). Formulated ivermectin (Noromectin) was from Norbrook Laboratories (Newry, Ireland). Compounds were dissolved in dimethyl sulfoxide (DMSO) and appropriate dilutions were produced to perform the assays.

### 2.2. Synthesis

- General Chemical Techniques

Reagents and solvents (dichloromethane, chlorobenzene, methanol, hexane, ethyl acetate) were used as purchased, without further purification. TLC was performed on precoated silica gel polyester plates (0.25 mm thickness) using a UV fluorescence indicator 254 (Polychrom SI F254). Microwave reactions used a Monowave 300 (Anton Paar). Synthetized compounds were characterized by ^1^H nuclear magnetic resonance (NMR) and ^13^C NMR using a Bruker SY spectrometer (Ettlingen, Germany) at 400/100 MHz or a Varian Mercury (Palo Alto, CA, USA) 400/100 MHz spectrometer and high-resolution MS using a hybrid QSTAR XL quadrupole/time of flight spectrometer (Foster City, CA, USA).

- Synthesis

Synthesis of *N*-(6-methylbenzo[*d*]thiazol-2-yl)nicotinamide (**20**)


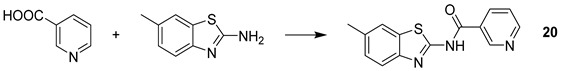


First, 100 mg (0.812 mmol) of nicotinic acid and 133 mg (0.812 mmol) of 6-methylbenzo[*d*]thiazol-2-amine were suspended in 12 mL of dry chlorobenzene. Phosphorus trichloride (0.406 mmol) was added, and the reacting mixture was heated in the microwave reactor for 15 min at 130 °C using infrared flask–surface control of the temperature. The reaction mixture was washed with water and filtered to yield 56 mg. Crystallization in a mixture of EtOAc/CH_2_Cl_2_/Hexane gave 32 mg (0.119 mmol, 14.6%) of *N*-(6-methylbenzo[*d*]thiazol-2-yl)nicotinamide (**20**).

*N*-(6-methylbenzo[*d*]thiazol-2-yl)nicotinamide (**20**). ^1^H-NMR (DMSO-*d*_6_): 2.40 (3H, *s*); 7.26 (1H, *d*, *J* = 8.0 Hz); 7.58 (1H, *dd*, *J* = 4.8 and 8.0 Hz); 7.65 (1H, *d*, *J* = 8.4 Hz); 7.78 (1H, *s*); 8.43 (1H, *d*, *J* = 8.0 Hz); 8.79 (1H, *d*, *J* = 4.0 Hz); 9.23 (1H, *s*). ^13^C-NMR (100 MHz, DMSO-*d*_6_): 21.5 (CH_3_); 120.2 (C); 121.9 (CH); 124.1 (CH); 128.1 (CH); 128.7 (C); 131.9 (C); 133.8 (C); 136.6 (CH); 149.7 (CH); 153.4 (CH); 158.6 (C); 165.5 (C). One tertiary carbon not observed. HRMS: Calculated for C_14_H_11_ON_3_SNa^+^ 292.0515, found 292.0510 (M + Na^+^). M.p. = 275 °C.

Synthesis of 6-chloro-*N*-(6-methylbenzo[*d*]thiazol-2-yl)nicotinamide (**21**)


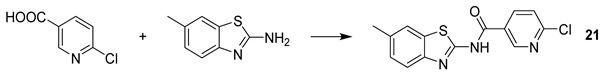


An excess of SOCl_2_ was added to 270 mg (1.71 mmol) of 6-chloronicotinic acid and stirred at 70 °C for 72 h. Then, the mixture was evaporated and the residue was added to a solution of 200 mg (1.22 mmol) of 6-methylbenzo[*d*]thiazol-2-amine and triethylamine (1 mL) in 10 mL of CH_2_Cl_2_. The reaction mixture was stirred at room temperature for 24 h and filtered, obtaining 295 mg (0.97 mmol, 79%) of 6-chloro-*N*-(6-methylbenzo[*d*]thiazol-2-yl)nicotinamide. Crystallization in MeOH/CH_2_Cl_2_ 95:5 yielded 67 mg (0.22 mmol, 10%) of 6-chloro-*N*-(6-methylbenzo[*d*]thiazol-2-yl)nicotinamide (**21**).

6-Chloro-*N*-(6-methylbenzo[*d*]thiazol-2-yl)nicotinamide (**21**). ^1^H-NMR (DMSO-*d*_6_): 2.41 (3H, *s*); 7.28 (1H, *dd*, *J* = 1.7 and 8.2 Hz); 7.64 (1H, *d*, *J* = 8.2 Hz); 7.71 (1H, *d*, *J* = 8.4 Hz); 7.79 (1H, s); 8.46 (1H, *dd*, *J* = 2.7 and 8.2 Hz); 9.06 (1H, *d*, *J* = 2.7 Hz); 13.10 (NH, *s*). ^13^C-NMR (100 MHz, DMSO-*d*_6_): 21.5 (CH_3_); 110.0 (C); 122.0 (CH); 124.8 (CH); 128.2 (CH); 133.9 (C); 140.0 (CH); 150.4 (CH); 154.2 (C). One tertiary carbon and three quaternary carbons not observed. HRMS: Calculated for C_14_H_12_ON_3_ClS^+^ 304.0306, found 304.0306 (M + H^+^). M.p. = 269 °C.

Synthesis of *N*-(6-methylbenzo[*d*]thiazol-2-yl)-6-(4-methylpiperazin-1-yl)nicotinamide (**22**).


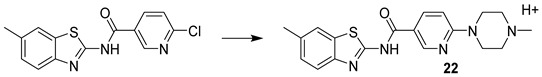


*N*-methylpiperazine (1 mL) was added to 105 mg of 6-chloro-*N*-(6-methylbenzo[*d*]thiazol-2-yl)nicotinamide (0.29 mmol) and heated at 75 °C for 30 min. Then, the reaction mixture was poured into cold water and extracted with CH_2_Cl_2_, obtaining 99 mg. Crystallization in MeOH/CH_2_Cl_2_ 99:1 gave 28 mg (0.08 mmol, 28%) of **22**.

*N*-(6-methylbenzo[*d*]thiazol-2-yl)-6-(4-methylpiperazin-1-yl)nicotinamide (**22**). ^1^H-NMR (CDCl_3_): 2.34 (3H, *s*); 2.45 (3H, *s*); 2.49 (4H, *m*); 3.68 (4H, *m*); 6.55 (1H, *d*, *J* = 9.1 Hz); 7.13 (1H, *dd*, *J* = 1.7 and 8.3 Hz); 7.36 (1H, *d*, *J* = 8.3 Hz); 7.61 (1H, *d*, *J* = 1.7 Hz); 8.00 (1H, *dd*, *J* = 2.5 and 9.1 Hz); 8.79 (1H, *d*, *J* = 2.5 Hz); 10.94 (1H, s). ^13^C-NMR (100 MHz, DMSO-*d*_6_): 21.4 (CH_3_); 44.5 (2) (CH_2_); 46.1 (CH_3_); 54.7 (2) (CH_2_); 105.5 (CH); 116.0 (C); 120.1 (CH); 121.2 (CH); 127.5 (CH); 132.2 (C); 133.8 (C); 136.9 (CH); 145.8 (C); 149.3 (CH); 158.7 (C); 160.5 (C); 164.1 (C). HRMS: calculated for C_19_H_22_ON_5_S^+^ 368.1540, found 368.1533 (M + H^+^). M.p. = 225 °C.

### 2.3. Animals, S. venezuelensis Life Cycle Maintenance, and Ethics Statement

Animal procedures complied with the European Union (Di 2010/63/CE) and the Spanish (RD53/2013) regulations on animal experimentation. The University of Salamanca’s Ethics Committee approved the procedures followed in this study (Protocols: CEI 1062 and CEI 1080). Male Wistar rats weighing 80–120 g, from the Animal Experimentation facilities of the University of Salamanca (Registration No. PAE/SA/001), and female CD1 mice (Charles River, Lyon, France) weighing 25–30 g were used for the maintenance of the *S. venezuelensis* life cycle and for in vivo experiments. Animals were kept in standard polycarbonate and wire cages with regular 12 h light–dark periods and temperatures of 20 and 24 °C and had free access to standard laboratory chow and water. At the end of the experimentation, or if the animals presented any deterioration in their health status, they were humanely euthanized by the intraperitoneal injection of a lethal dose of pentobarbital (100 mg/kg). All efforts were made to minimize animal suffering.

The *S. venezuelensis* strain, from the Department of Parasitology, Federal University of Minas Gérais (Brazil), was maintained by serial passage in Wistar rats at the Parasitology Department of the University of Salamanca. Wistar rats were subcutaneously infected with 12,000 third-stage larvae (L3) in 500 µL of phosphate-buffered saline (PBS) using a 23-gauge needle syringe. Feces from infected rats (5–21 days p.i.) were collected and cultured with vermiculite and water at 28 °C for 4 to 7 days in a humid atmosphere. L3s were recovered using a Baermann apparatus, washed twice with distilled water, and used for new infections or in vitro experiments.

### 2.4. In Vitro Activity against L3

Baerman-obtained *S. venezuelensis* L3s were rinsed twice with distilled water and counted, and 100 to 150 larvae were placed per well in 96-well flat-bottom culture plates. Larvae were incubated at 28 °C for 30 min to allow adaptation and treated with compounds in the range of 0.5–40 µM for 72 h at 28 °C in a humid atmosphere. Mortality was assessed as the lack of any movement detected during 1 min of observation under an inverted microscope (40×), at 24, 48, and 72 h after treatment. The ability to stain the larvae was pointed out when observed; at 72 h, larvae were collected and washed two times with distilled water and placed in a new well to remove the background color and photographed. Pictures were also taken at 24 and 48 h without cleaning the larvae so as not to interfere with the 72 h readout of the experiment. *S. venezuelensis* larvae were incubated in water with 1% DMSO or 10 µM ivermectin as negative and positive controls, respectively; 1% DMSO was the concentration used for the experiments at the compounds’ maximum concentrations. All experiments were conducted in triplicate and at least three different times. The antiparasitic activity of the compounds was expressed as the concentration that inhibited the motility of half of the larvae (immobilization concentration 50, IC_50_).

### 2.5. In Vitro Activity against Adult Females

Seven to nine days after infection, the mice or rats were euthanized and the small intestine was removed, sliced longitudinally, minced, and placed in a sedimentation cup wrapped in 8 layers of gauze in phosphate-buffered solution (PBS) for two hours at 37 °C. Parasitic females were collected from the sediment and washed twice with saline solution (NaCl 0.15 mol/L) and twice with Roswell Park Memorial Institute 1640 (RPMI 1640) (Gibco, Whaltman, MA, USA) supplemented with 10% heat-inactivated FBS, 100 µg/mL streptomycin, and 100 U/mL penicillin (Gibco, Whaltman, MA, USA) to avoid contamination during the in vitro assays. After collection, females were placed in 96-well plates with supplemented RPMI medium and kept at 37 °C. We tested three different female population densities to explore the effects on the measured activity: 1–3 females, 10–15 females, and 50–70 females per well. At 24 h after plating, the compounds were applied at 10 µM and the anti-parasitic activity was evaluated at 1, 24, 48, and 72 h after treatment based on movement for 30 s observation periods under the microscope (40×). They were classified into four categories: healthy, slightly affected (slower movement), affected (moderate decrease in movement or abnormal movement), and death (absence of movement). All experiments were conducted in duplicate and at least three different times.

### 2.6. Cytotoxicity of the Compounds

A cytotoxicity test was performed using the HeLa (human cervical cancer) cell line according to the MTT method. Cells were from the ATCC (Manassas, VA, USA). They were cultured at 37 °C in sub-confluent conditions under a 95% humidified atmosphere and 5% CO_2_ in Dulbecco’s Modified Eagle Medium (DMEM) (Gibco, Whaltman, MA, USA) supplemented with 10% heat-inactivated FBS, 2 mM L-glutamine, 100 U/mL penicillin, and 100 µg/mL streptomycin. HeLa (1.5 × 10^3^) cells were seeded in 96-well plates and incubated in the presence of the compounds in the range of 0.1–250 µM when the solubility of the compound allowed it. The antiproliferative activity was measured in triplicate after 72 h using the MTT (3-(4,5-dimethylthiazol-2-yl)-2,5-diphenyltetrazolium bromide) Cell Proliferation Kit (Roche, Boston, MA, USA), following the manufacturer’s specifications. Each experiment was repeated at least three times in triplicate to calculate the inhibitory concentration 50 (IC_50_) values, the drug concentration required to inhibit 50% of the cell growth with respect to the untreated control. The selectivity index (SI = HeLa IC_50_/L3 IC_50_) for each compound was calculated to compare the antiparasitic activity with its respective human cell cytotoxicity.

### 2.7. Calculation of Properties

The structures of the dyes and commercial products were searched in PubChem, from which the SMILE codes were obtained. The synthesized compounds were drawn in ChemDraw and their SMILE codes were obtained. The codes were used in the in silico prediction software eNTRy-way [16] and SwissADME [28].

### 2.8. In Vivo Activity of Dyes

**Scheme 1:**
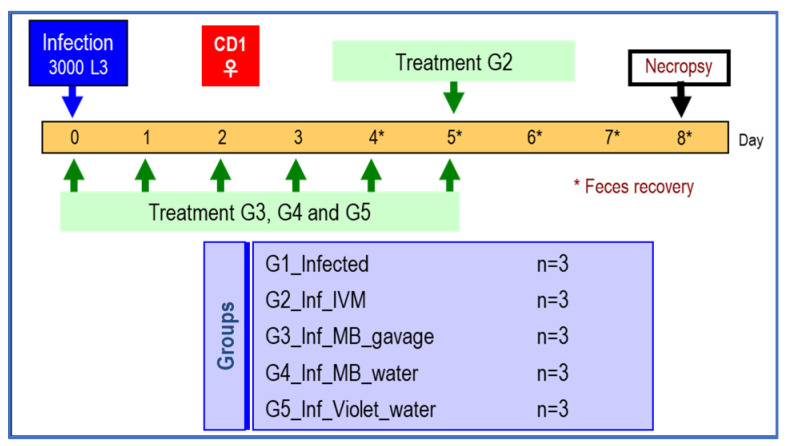
In vivo procedure. Mice were infected at day 0 and treated at days 0, 1, 2, 3, 4, and 5 p.i. Feces were recovered at days 4, 5, 6, 7, and 8 p.i.

Methylene Blue (**1**) and Crystal Violet (**3**) were tested in *S. venezuelensis* experimental infections of mice in a pilot study. Fifteen CD1 mice were randomly distributed in seven experimental groups with 3 mice per group as follows: Group 1, infected control; Group 2, infected and treated with ivermectin (Noromectin); Group 3, infected and treated with Methylene Blue intragastrically; Group 4, infected and treated with Methylene Blue in drinking water; and Group 5, infected and treated with Crystal Violet in drinking water (see Scheme 1).

Ivermectin was administered as a single oral dose of 0.2 mg/kg on day 5 p.i. Methylene Blue (group 3) was administered at 20 mg/kg/day from the infection day to day 5 p.i. Methylene Blue (group 4) and Crystal Violet (group 5) were dissolved in the drinking water with the estimation that the mice would ingest 20 mg/kg/day from the day of infection to day 5 p.i. and, after this, the water was replaced with fresh water. All animals were infected by subcutaneous injection with 3000 L3s of *S. venezuelensis* resuspended in PBS. Individual fecal samples from days 5 to 8 p.i. were collected and preserved in a 10% formalin-buffered solution, and eggs were counted in triplicate samples, measuring the numbers of eggs in a determined volume. The eggs per gram was reported. The recovery of parasite females was performed as described above. The mean was determined for the eggs per gram (EPG) of feces and parthenogenetic female counts. The color of the feces and urine in the beds was recorded.

### 2.9. Statistical Analysis

Statistical analysis was performed using the R Studio 2024.04.2-764 software (R Core Team, 2024). The IC_50_ values were calculated using the R package “drc” [29] with a 4-parameter log-logistic model. Plots and figures were created using the package “ggplot2” [30].

## 3. Results

### 3.1. In Vitro Activity of Dyes against L3

A total of 15 dyes were tested against *S. venezuelensis* L3s at a 20 µM concentration. The activity was determined by the number of larvae lacking motility relative to the total number in the well after 1 min of light excitation at 24, 48, and 72 h after treatment. Compounds were classified as active when there was an increase of at least 30% in immobile L3s compared to the negative controls. For those dyes showing activity, the IC_50_ values were determined when possible. Table 1 shows a summary of the results.

**Figure 1:**
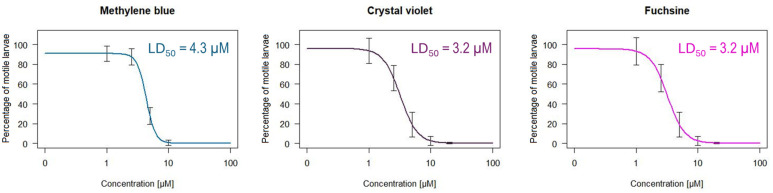
Dose–response curves and calculation of IC_50_ values for Methylene Blue, Fuchsine, and Crystal Violet after 72 h of treatment.

The most potent dyes were Fuchsine (IC_50_ = 3.2 µM) (Figure 1), Crystal Violet (IC_50_ = 3.2 µM) (Figure 1), Methylene Blue (IC_50_ = 4.3 µM) (Figure 1), propidium iodide (IC_50_ = 4.3 µM), and Brilliant Cresyl Blue (IC_50_ = 6.8 µM), with IC_50_ values close to that of ivermectin (IC_50_ = 1.5 µM) and at least 10-fold better than that of edelfosine (IC_50_ = 49.6 µM) [31], albendazole, and mebendazole (no activity at 40 µM). The IC_50_ values progressively decreased (i.e., became more potent) over time for all active dyes, with some of the actives at 72 h showing IC_50_ values above the selected threshold of 20 µM at shorter times. 

Azur–Eosin–Methylene Blue and Trypan Blue only showed activity after 72 h of treatment, with IC_50_ values of 17.6 µM and 27.9 µM, respectively. The Azur–Eosin–Methylene Blue combination resulted in a worse IC_50_ value than Methylene Blue alone (16.67 µM vs. 4.35 µM respectively). Trypan Blue, Sudan III, Hematoxylin, Congo Red, Carmine, Indigo Carmine, Methyl Orange, Eriochrome Black T, and fluorescein diacetate did not show significant activity against the L3s. The five dyes (**1**–**5**) with high activity against L3s bore positive charges, while neutral, zwitterionic, and negatively charged compounds did not significantly reduce L3s’ motility after 72 h of treatment. The only neutral molecule with activity against L3s was ivermectin.

**Figure 2:**
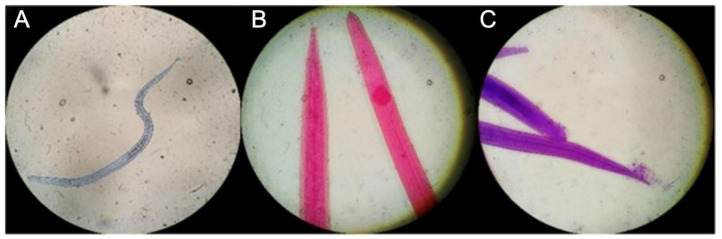
Images of L3s stained with Methylene Blue (100×) (**A**), Fuchsine (400×) (**B**), and Crystal Violet (400×) (**C**). L3s were treated with each compound at 10 µM and photographed after 24 h.

All dyes that stained the L3s showed antiparasitic activity (Figure 2).

### 3.2. Cytotoxicity of Dyes against HeLa Cell Line

Active dyes against *Strongyloides* were tested for antiproliferative activity after 72 h of treatment against HeLa cells to assess mammal cytotoxicity. Brilliant Cresyl Blue (>250 µM) and Trypan Blue (>10 µM) did not show any cytotoxicity at the tested concentrations. Azur–Eosin–Methylene Blue (8.4 µM), Methylene Blue (1.1 µM), Fuchsine (1.0 µM), and Crystal Violet (1.0 µM) showed cytotoxicity values in the low micromolar range. The IC_50_ values of the control drugs were as follows: ivermectin (5.9 µM), albendazole (0.3 µM), and mebendazole (0.2 µM). The SI values for these compounds were ranked as follows: Brilliant Cresyl Blue (SI > 36.8), ivermectin (SI = 3.9), Fuchsine (SI = 0.31), Crystal Violet (SI = 0.31), Methylene Blue (SI = 0.26), albendazole (SI < 0.0075), and mebendazole (SI < 0.005).

### 3.3. In Vitro Activity of Dyes against Adults

Throughout the parasitic life cycle, parasitic females live embedded in the small intestinal mucosa, producing eggs. Compounds that were active against the L3s (1–5) and the reference compounds of ivermectin, mebendazole, and albendazole were assayed against the female stage of *S. venezuelensis* at a 10 µM concentration at different time points, with a population density of 50–70 worms per well (see Table 2).

Among the dyes, only Methylene Blue, Brilliant Cresyl Blue, and propidium iodide significantly affected the parasitic females. Methylene Blue achieved the best results (IC_50_ values of 6.02 ± 0.9 µM), which were even better than those of the reference drugs. The effects of the dyes and the benzimidazoles on the adult females increased over time, while, for ivermectin, it was more pronounced at the shortest time points, with a subsequent decay after 48 h.

**Figure 3:**
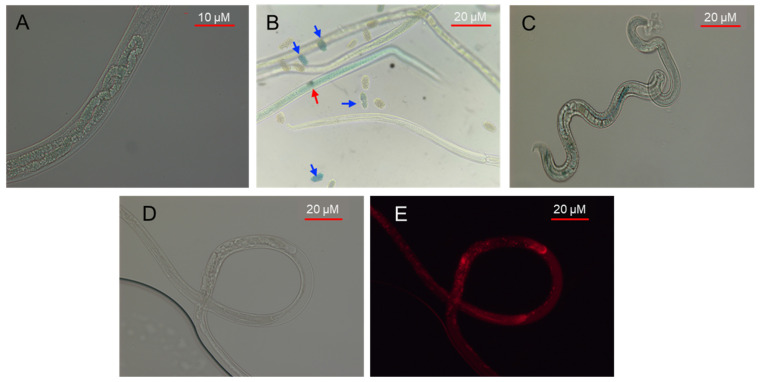
Stained intestine inside an adult of *S. venezuelensis* treated with Methylene Blue at 10 µM for 72 h (**A**). Stained *S. venezuelensis* adults (red arrow) and eggs (blue arrows) treated with Methylene Blue at 10 µM for 72 h (**B**). Stained intestine in adults treated with Brilliant Cresyl Blue at 10 µM for 72 h (**C**). Adults treated with propidium iodide at 10 µM for 72 h, showing diffuse and intense points in inner structures ((**D**) white field and (**E**) fluorescence).

At this stage, Methylene Blue clearly stained the adult females at 48 h post-treatment (Figure 3A), with some worms being slightly stained as soon as 24 h post-treatment. We also observed that some eggs were instantly stained after adding Methylene Blue, while others with a similar morphology were not, even throughout the whole experiment (Figure 3B). Brilliant Cresyl Blue stained adult females after 24 h in some localized structures inside the parasite (Figure 3C). In both cases, the staining was much less noticeable than for the L3 stage. Crystal Violet and Fuchsine did not stain the adult parasites. Regarding propidium iodide, the staining was more evident in those females that were dead due to handling, which is also in line with its usual application as a vital dye (only cells with permeable membranes, usually dead ones, are stained); living worms were not strongly stained, but fluorescent eggs could be observed inside them (Figure 3D,E) (Supplementary Material Video S1).

Ivermectin provoked noticeable spasmodic movement in the adult females after 1 h of incubation, which lessened after 48 h. Females treated with albendazole or mebendazole adopted abnormal shapes (more elongated and less curved than in the healthy control) and slightly reduced movement, starting after 48 h of treatment (Supplementary Material Figure S2).

The experiments were conducted using different numbers of parasites per well (ranging from 50–70 to 10–15 and 1–3) (Supplementary Material Table S2). The estimated potencies improved when decreasing the number of adults per well, especially at the lowest parasite numbers. However, none of the inactive dyes in the high-larvae-content experiment became lethal to the larvae or severely affected them in the lower-content experiments.

### 3.4. In Vivo Activity of Dyes

**Figure 4:**
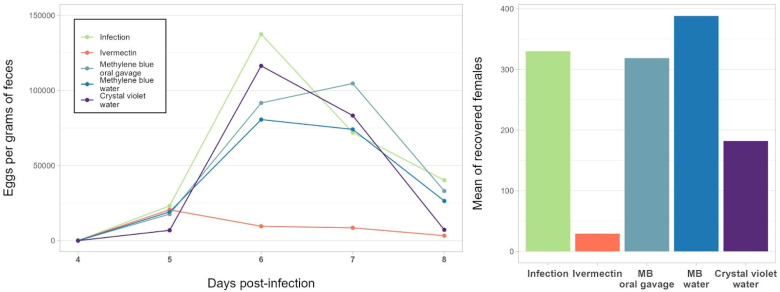
Eggs per gram of feces through infection and number of females recovered at day 8 p.i. in mice infected with 3000 L3s and treated with Methylene Blue administered by oral gavage and Methylene Blue and Crystal Violet provided ad libitum in drinking water.

After yielding promising results in the in vitro studies with the L3s and parthenogenetic females, Methylene Blue (**1**) and Crystal Violet (**3**) were assayed in a murine experimental infection model with 300 L3s and using the effective treatment of ivermectin as a control. Egg seeding in feces for five days (days 4 to 8 p.i.) and parthenogenetic females at day 8 p.i. were used to determine the nematocidal activity. Eggs in feces started to appear at day 5 p.i. for all groups (6861 ± 5585 to 23,141 ± 15,741), peaking at day 5 p.i. (ivermectin, 20,534 ± 15,961), day 6 p.i. (untreated, MB and Crystal Violet in drinking water, 80,617 ± 31,706 to 116,364 ± 82553), or day 7 p.i. (MB gavage, 104,635 ± 23,710) and decreasing at day 8 p.i. (Figure 4), but there were no statistically significant differences. Neither of the treated groups (187 ± 101 to 388 ± 54 parthenogenetic females) exhibited a significant difference (Kruskal–Wallis H Test: χ^2^ = 6.9667, df = 4, P = 0.1377) compared to the infected control group (330 ± 118). The ivermectin-treated group showed reductions (29 ± 12.3), but they were not statistically significant. A large reduction in the number of parthenogenetic females was noticed in the ivermectin-treated control group (29 females recovered per mouse on average, compared to 340 in the infection group—a 91.5% reduction). Regarding the dye-treated groups, there was only a reduction in the Crystal Violet one (an average of 182 females recovered per mouse—a 46.5% reduction) (Figure 4). 

To determine the uptake and clearing of the dyes, the color of the feces and urine was assessed. The feces in groups 3 and 4 were stained within hours on day 1, and the color disappeared on day 6 (24 h after the last treatment), while the feces in group 5 were not stained until day 3 and the staining lasted longer than for groups 3 and 4, disappearing at day 8. Urine staining only occurred for groups 3 and 4.

### 3.5. Structural Analysis of Active Dyes

We analyzed the chemical structures of the active dyes to find common structural patterns that could help in the definition of entry rules for *Strongyloides* L3s and adult states. The compounds were classified as positively charged, negatively charged, zwitterions, or neutral based on their chemical structures (Table 1) (Supplementary Material Figure S1). The properties that have been shown to determine uptake for Gram-negative bacteria were calculated (Supplementary Material Table S3) [16], i.e., the molecular formula, molecular weight, number of rotatable bonds, globularity, average distance to the plane of best fit (PBF), and functional group assessment (presence/absence of primary, secondary, or tertiary aliphatic amines or ammonium groups). Additional chemical properties were calculated with SwissADME (Supplementary Material Table S2): the molecular weight, number of heavy atoms, number of aromatic atoms, fraction of sp3 carbons, number of rotatable bonds, number of hydrogen-bond acceptors and donors, molar refractivity, topological surface area, different lipophilicity (logP) and solubility estimates, skin permeability coefficient (Kp), sensitivity to glycoprotein efflux pumps (Pgp substrate), inhibition of several CYP isoforms, number of violations of drug-likeness rules (Lipinski, Ghose, Veber, Egan, and Muegge), the related Abbot Bioavailability Score, Pan Assay Interference compounds (PAINS) (i.e., frequent hitters or promiscuous compounds), Brenk (list of 105 fragments that are putatively toxic, chemically reactive, metabolically unstable, or responsible for poor pharmacokinetics) alerts, violations of leadlikeness, and synthetic accessibility. These properties are used by medicinal chemists in drug discovery efforts, as they can serve as predictors of the pharmacokinetic and pharmacodynamic properties of drug candidates. The color coding of the value properties and a comparison with the active/inactive dyes in Supplementary Material Tables S2 and S3 allowed us to select properties that condition activity; these were low globularity for adult parthenogenetic females and a positive charge for adults and L3s.

### 3.6. Design and Synthesis of Modified Compounds with Potential Anti-Strongyloides Activity

We selected those compounds from the tested in-house library that failed to show anti-strongyloides activity but fulfilled some of the structural rules deduced for dye uptake by L3 and adult females. The most frequent factor missing was the presence of a positively charged amino group. We therefore chose one of the compounds (**20**) with good synthetic access to a positively charged analog (**22**) and synthesized and purified it. The chemical synthesis consisted of the formation of the amide bond between 6-chloropicolinic acid and 2-amino-6-methyl-benzothiazole, and the subsequent nucleophilic aromatic substitution reaction of the chlorine atom with *N*-methylpiperazine led to **22** in a good yield.

### 3.7. In Vitro Activity of Positively Charged Compound 22 against S. venezuelensis

We tested our new positively charged compound (**22**) along with its uncharged version at a 20 µM concentration against the L3 and adult female stages (Table 3). Positively charged compound **22** caused a 100% L3 mortality rate at 20 µM after 72 h and had a calculated LC_50_ of 8.6 µM, while the uncharged analog **20** did not show any activity at 20 µM. Time course experiments showed a potency increase over time, as previously observed for the active dyes and the benzimidazoles and in contrast to ivermectin. Both compounds were also tested against adults at 10 µM, with compound **22** slightly affecting but not killing females after 72 h of treatment. The uncharged precursor did not show any potency. Finally, the compounds were assayed against HeLa cells using the MTT method, showing no antiproliferative activity at 10 µM.

## 4. Discussion

Strongyloidiasis is likely the most neglected amongst the neglected tropical diseases (NTDs); it is a disease that affects disadvantaged and marginal populations. The appearance of resistant parasites, the reliance on only three drugs to control it, and the inefficiency of the available treatments in immunocompromised patients necessitate the development of new drugs. However, it has been disregarded in international helminth control programs due to the clear underestimation of its global burden, which limits funding and reduces the drug discovery campaigns even beyond other NTDs [7].

Drug discovery regarding NTDs is plagued with difficulties, and strongyloidiasis is not an exception [14]. Drug screening campaigns encounter challenges due to funding, the complex biology, and the difficult access to in vitro screens for the therapeutically relevant life cycle stages, as well as the limited knowledge of the parasite’s biology and the unknown requirements for drugs to be efficient. We started a screening campaign to discover new compounds that are active against the therapeutically relevant L3 stage of the model *S. venezuelensis* and found that the rate of discovery of actives lagged behind those of related parasites. Taking Gram-negative bacteria as a reference for low-success drug discovery campaigns, we reasoned that drug uptake and accumulation might be the reasons for this lack of success. The study of drug uptake and accumulation in multicellular systems is more complex than in unicellular organisms, as different cells and tissue types can behave differently and the external coatings might impose additional constraints. We therefore resorted to dyes, as they provide an efficient means of assessing uptake and distribution within parasites, and started screening them against *S. venezuelensis* in vitro, examining their uptake and the effects on their viability.

The dyes showed a significant success rate against L3s, with 5 out of 15 dyes showing activity at a threshold concentration of 20 µM. The IC_50_ evaluations at 24, 48, and 72 h showed potency at 72 h that was comparable to that of the reference drug ivermectin (IV) and better than that of edelfosine or the benzimidazoles, albendazole or mebendazole. These dyes have yielded some of the best IC_50_ values against L3s in in vitro assays so far. Azur–Eosin–Methylene Blue and Trypan Blue also showed some activity. However, the former is a combination of Methylene Blue with other dyes, and its activity is worse than that of Methylene Blue alone, suggesting that its activity comes mainly or fully from this dye. Only the active dyes stained the L3s, whereas inactive dyes did not. Furthermore, staining occurs earlier and at lower or similar concentrations to those affecting L3 motility. This suggests that the crossing of the *S. venezuelensis* L3 cuticle to allow uptake and accumulation governs the activity. This is of extreme importance, and, to the best of our knowledge, this study is one of the first to address this topic, as conventional approaches to the discovery of anti-helminthic drugs usually fail to consider uptake.

The success rate for the dyes is lower in the adult stage, where only Methylene Blue effectively kills the parasite below a 10 µM concentration after 48 h. Out of the five active dyes against the L3 stage, only two achieve staining and have significant effects on the adult females, indicating that this stage is even less permeable than the L3. The potency of Methylene Blue against the females is quite superior to that of the reference drugs, ivermectin and the benzimidazoles (IC_50_ values above 229 µM and 760 µM, respectively, never achieving the complete absence of movement at these concentrations [32,33]). Ivermectin at the tested concentration provoked unnatural movement in the females shortly after treatment, in line with its mechanism of action. These results are consistent with the increase in the velocity of movement previously reported for *S. venezuelensis* L3s treated with low ivermectin concentrations [34,35].

Moreover, as seen for the L3s, the potency of the dyes increases over time, similarly to that observed for the benzimidazoles and contrary to the trend observed for ivermectin, which shows the strongest effect at the shortest timepoint and then exhibits a continuous potency decrease.

We explored the effect of the number of adult females on the results of the screening experiments and found that small numbers of adults led to overestimations of the potency of the assayed compounds. This observation is important when comparing drugs’ effects from different studies and should be considered when new screening campaigns are planned, especially for those diseases caused by hundreds or thousands of parasites in the host, such as strongyloidiasis. Often, antiparasitic studies, especially those on helminths, are conducted with low parasite numbers due to the difficulties in access to larger numbers, but this limits their significance [33].

The high potency in the two therapeutically relevant stages suggests that the most potent dyes might be possible treatments for strongyloidiasis. To obtain an estimate of their potential toxicity, the antiproliferative effects of the dyes against the human cervix cancer cell line HeLa were evaluated. They showed antiproliferative activity at the micromolar range, which might indicate toxicity towards the host. However, many of the tested dyes find widespread use in the food industry and medicine, and their safety has been widely proven [21,26,27,36,37].

Methylene Blue and Crystal Violet were tested in a pilot in vivo experiment but were unable to halt or prevent the infection, achieving only a decrease in the number of parthenogenetic females and not in the number of EPGF in the group treated with Crystal Violet. The staining of the feces and urine suggests that the drugs are eliminated by the time that the females reach the intestine, their final destination, thus limiting the efficacy of the dyes in reducing the infection [21,38,39]. The slow-acting nature of the compounds, which need hours or days to reach significant antiparasitic effects in vitro, combined with this fast elimination rate, suggests that they may fail to reach and maintain an effective concentration that could successfully act against the parasite in the gut, even when administered in the drinking water in an attempt to increase the contact time. Previous work in our group showed that the use of formulations significantly improves the effectiveness of ivermectin. This opens the door to the development of formulations to enhance their ADMET properties and achieve sufficient and sustained concentrations to affect the parasite. Considering the poor pharmacokinetics of the active dyes, which rule them out as direct treatment options, we focused our attention on deriving rules for uptake and accumulation within *Strongyloides* regarding their chemical structures, which could be applied to the design and discovery of non-dye compounds.

Analyzing the calculated properties of the tested dyes, L3 active dyes have intermediate TPSAs between 9 and 80 and consensus logP values between 0 and 2, indicating a relatively polar nature but not exceedingly so, with values compatible with passive membrane crossing. However, these properties are also shared with inactive, non-penetrating dyes. The difference between the two types is that only positively charged ones seem to pass through the L3 cuticle; ivermectin, which works precisely by affecting glutamate-gated chloride channels [34], is the only neutrally charged compound with activity against L3s. In a similar manner, it has been observed that cationic detergents are more effective in extracting proteins from the cuticle of *Strongyloides* and other nematodes than non-ionic, zwitterionic, or anionic detergents [40,41]. Other organisms with thick cell walls, such as Gram-negative bacteria, have also shown a preference for the uptake of positively charged compounds. In these organisms, scaffolds containing a primary amine are favored for entry and accumulation [17,42]. Moreover, recent research that focused on Pseudomonas aeruginosa reinforces this assertion and expands it to encompass any positive charge, rather than only primary amines [43]. This requirement has been suggested to underlie the fact that only a small portion of the compounds present in chemical libraries show activity and thus pass the barrier [44]. The adult females seem to be even more restrictive than the L3s, with fewer dyes being able to stain and affect them. The structural comparison of the two active and three inactive dyes against the adult stage among the L3 active dyes (thus carrying a positive charge as a prerequisite) shows that the first group has lower globularity (are more linear) than the inactive ones. These chemical rules for uptake in the therapeutically relevant stages provide a design principle for new drugs against strongyloidiasis.

As a proof of concept, we designed, synthesized, and assayed a positively charged analog of a low-globularity neutral compound (**20**), previously tested as a component of our chemical libraries, which showed no in vitro activity at 20 µM at 72 h. The charged analog **22** achieved an IC_50_ of 8.6 µM against the L3 stage and slightly affected the adult females at 10 µM after 72 h of treatment. The introduction of a positive charge on an inactive analog restored the activity, thus suggesting that drug uptake was responsible for the different behavior. The placement of the positive charge has not been optimized, as good synthetic access was pursued in this proof of concept. Future work will focus on exploring the effects on the activity of different positively charged groups and different attachment points to the skeletons. Successful penetration will in turn allow us to establish structure–activity relationships and gain information on the target preferences for the compounds. The extension of these modifications to our chemical libraries is expected to improve our success rates. These findings suggest means of improving the possibility of finding active compounds and configuring the chemical libraries for screening campaigns, prioritizing positively charged compounds and placing greater emphasis on guaranteeing access to the parasite.

## 5. Conclusions

In the search for new treatments for strongyloidiasis, we achieved very low success rates, which suggested that most compounds failed to access the interior of the parasite. Using dyes to ascertain uptake, we found that some dyes had promising in vitro activity against the therapeutically relevant stages, but they failed in a mouse model of the disease due to pharmacokinetic issues. New formulations that could prolong the contact time with the parasite in the gut might transform these dyes into possible alternatives for the treatment of strongyloidiasis, as many of them have already been used in the clinic, and a repurposing strategy could then be achieved [14].

With the structural information gathered from the uptake of the active dyes, we have established drug properties that impact the uptake of chemical compounds by different life cycle stages of *Strongyloides*: a positive charge and low globularity endow compounds with the capability to access the interior of the parasite. These rules are similar to those found for drug uptake in Gram-negative bacteria—rules that allow the transformation of inactive into active compounds. We therefore selected one inactive compound that fulfilled the geometrical requirements for activity and equipped it with a positive charge. The new analog provides a proof of concept as it regained its antiparasitic activity. The rules for uptake can be applied to select chemical compounds for antiparasitic drug screening campaigns and to modify target-designed ligands that have failed to show activity, probably due to a lack of uptake.

These results provide new and important advances in the pursuit of new treatments against strongyloidiasis, an underestimated NTD that affects hundreds of millions of people and whose clinical treatment is insufficient. These findings could also be extended to other parasitic diseases with a need for new therapeutic approaches.

## Figures and Tables

**Table 1:** Activity of the dyes and the reference drugs screened at 20 µM; immobilization concentration 50 values of cultured L3s (IC_50_) at 24, 48, and 72 h; L3 staining ability; lowest staining concentration (Conc); and charge of each molecule. N.d., not determined.

Number	Compound	Screening 20 µM 72 h	IC_50_ 24 h (µM)	IC_50_ 48 h (µM)	IC_50_ 72 h (µM)	Staining 72 h (Conc)	Charge
**1**	**Methylene Blue**	Active	10.4 ± 3.2	6.8 ± 2.3	4.3 ± 0.3	Yes (1 µM)	Positive
**2**	**Brilliant Cresyl Blue**	Active	14.0 ± 4.1	9.6 ± 0.6	6.8 ± 0.7	Yes (2.5 µM)	Positive
**3**	**Crystal Violet**	Active	14.5 ± 2.4	7.7 ± 1.2	3.2 ± 0.4	Yes (2 µM)	Positive
**4**	**Fuchsine**	Active	7.4 ± 1.3	6.8 ± 0.9	3.2 ± 0.4	Yes (2 µM)	Positive
**5**	**Propidium** **Iodide**	Active	14.5 ± 4.6	9.8 ± 3.2	4.3 ± 0.6	Yes (3 µM)	Positive
**6**	**Azur** **–** **Eosin** **–** **Methylene Blue**	Active	N.d. >20	N.d. >20	16.7 ± 1.6	Yes (20 µM)	Positive–Negative– Positive
**7**	**Trypan Blue**	Active	N.d. >20	N.d. >20	27.9 ± 3.3	Yes (20 µM)	Negative
**8**	**Sudan III**	No	-	-	N.d. (>40)	No	Neutral
**9**	**Hematoxylin**	No	-	-	N.d. (>40)	No	Neutral
**10**	**Congo Red**	No	-	-	N.d. (>40)	No	Negative
**11**	**Carmine**	No	-	-	N.d. (>40)	No	Negative
**12**	**Indigo Carmine**	No	-	-	N.d. (>40)	No	Negative
**13**	**Methyl Orange**	No	-	-	N.d. (>40)	No	Negative
**14**	**Eriochrome Black T**	No	-	-	N.d. (>40)	No	Negative
**15**	**Fluorescein** **Diacetate**	No	-	-	N.d. (>40)	No	Neutral
**16**	**Ivermectin**	Active	2.1± 0.1	1.6 ± 0.3	1.5 ± 0.3	-	Neutral
**17**	**Edelfosine**	No	N.d. >40	N.d. >40	49.6 µM ± 0.5	-	Zwitterionic
**18**	**Albendazole**	No	-	-	N.d. (>40)	-	Neutral
**19**	**Mebendazole**	No	-	-	N.d. (>40)	-	Neutral

**Table 2:** Activity and staining ability of each molecule against parasitic females in cultures with 10 µM treatments after 1, 24, 48, and 72 h. Culture control was 1% DMSO as baseline. Healthy: normal and vivid movement and shape, no differences from the control. Slightly affected: slower movement or shape, different from the control. Affected: abnormally reduced movement and shape, altered from the control, and incompatible with normal life but not dead. Dead: immobile larvae.

Number	Compound	1 h	24 h	48 h	72 h (IC_50_/µM)	Staining (Time)
**1**	Methylene Blue	Healthy	**Affected**	**Death**	**Death** **(6.02 ± 0.9)**	**Yes (24 h)**
**2**	Brilliant Cresyl Blue	Healthy	Slightly affected	**Affected**	**Affected**	**Yes (24 h)**
**3**	Crystal Violet	Healthy	Healthy	Slightly affected	Slightly affected	No
**4**	Fuchsine	Healthy	Healthy	Healthy	Healthy	No
**5**	Propidium iodide	Slightly affected	Slightly affected	Slightly affected	**Affected/Some deaths**	**Yes (72 h)**
**16**	Ivermectin	**Affected**	**Affected**	Slightly affected	Slightly affected	**-**
**18**	Albendazole	Healthy	Healthy	Slightly affected	Slightly affected	**-**
**19**	Mebendazole	Healthy	Healthy	Slightly affected	Slightly affected	**-**

**Table 3:** Activity against L3 and parasitic females in cultures at 72 h of designed compound. Healthy: normal and vivid movement and shape, no differences vs. control; slightly affected: slower movement or shape, different from the control.

Number	Compound	Activity against L3 @20 µM 72 h	IC_50_ 72 h (µM)	Activity against Adults @10 µM 72 h
**20**	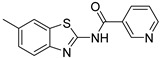	No	>20	Healthy
**22**	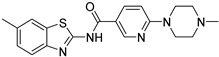	Yes	8.6 ± 2.3	Slightly affected

## Data Availability

The data underlying this study are available in the published article and its Supporting Information. Figure S1. Chemical structures of the dyes. Positively and negatively charged groups have been highlighted with pale blue and orange circles, respectively. Figure S2: Healthy parthenogenetic adult females of *Strongyloides venezuelensis* (A) and treated with 10 µM albendazole for 48 h. Table S1. Normalized numbers of parthenogenetic females and L3s in the in vivo experiments. EPGF = Eggs per gram in feces. Table S2. Properties calculated with SwissADME. The meaning of the parameters can be found at http://www.swissadme.ch/index.php (accessed on 10 January 2024). Table S3. Properties calculated with the entry rules page for the compounds (http://www.entry-way.org/pages/about (accessed on 10 January 2024)). Active compounds against L3s are indicated in yellow and a plus sign, and those against adult females with a cream plus. Rb = rotatable bonds. Glob = globularity. Pbf = average distance to the plane of best fit. Func_group = functional groups. Charge = total charge. Cells are colored by values to ease the comparisons. Table S4. Video S1. Adult female treated with fluorescein acetate under clear field and fluorescence microscope. Crude data are available from the authors upon request.

## Supplementary Materials

The following supporting information can be downloaded at https://www.mdpi.com/article/10.3390/pharmaceutics16091224/s1, Figure S1: Chemical structures of the dyes. Figure S2: Healthy parthenogenetic adult females of *Strongyloides venezuelensis* (A) and treated with 10 µM albendazole for 48 h (B). Table S1: Normalized numbers of parthenogenetic females and L3s in the in vivo experiments. Table S2: Properties calculated with SwissADME. Table S3: Properties calculated with the entry rules page for the compounds (http://www.entry-way.org/pages/about accessed on 10 January 2024). Table S4: Activities against different population densities (1–3 adults, 10–15 adults and 50–70 adults) at 72 hours with 10 µM treatments. Video S1: Adult female treated with fluorescein acetate under clear field and fluorescence microscope.
