# Supplementary material for: Deciphering Chemical Rules for Drug Penetration into Strongyloides
Source: Pharmaceutics. 2024 Sep 19;16(9):1224. doi: 10.3390/pharmaceutics16091224 (PMC11434980; doi:10.3390/pharmaceutics16091224)
Supplement: Supplementary file 1 [file pharmaceutics-16-01224-s001.zip › MMarin_SupplementaryMaterials_rev1.pdf]

Deciphering chemical rules for drug penetration into *Strongyloides*

Miguel Marín,[1,2,3] Javier Sánchez-Montejo,[3,4] Sergio Ramos Varela,[1,2,3] Antonio Muro,[3,4] Julio López-Abán,[3,4,*] Rafael Pélaez.[1,2,3,*]

SUPPLEMENTARY MATERIAL

Figure S1. Chemical structures of the dyes. Positively and negatively charged groups have been highlighted with pale blue and orange circles, respectively.

Figure S2: Healthy parthenogenetic adult females of Strongyloides venezuelensis (A) and treated with 10 μM albendazole for 48 hours.

Table S1. Normalized numbers of parthenogenetic females and L3s in the in vivo experiments. EPGF = Eggs per gram in feces.

Table S2. Properties calculated with SwissADME. The meaning of the parameters can be found at http://www.swissadme.ch/index.php

Table S3. Properties calculated with the entry rules page for the compounds (http://www.entry-way.org/pages/about). Active compounds against L3s are indicated in yellow and a plus sign, and those against adult females with a cream plus. Rb = rotatable bonds. Glob = globularity. Pbf = average distance to the plane of best fit. Func_group = functional groups. Charge = total charge. Cells are colored by values to ease the comparisons.

Table S4. Activities against different population densities (1-3 adults, 10-15 adults and 50-70 adults) at 72 hours with 10 μM treatments.

Video S1. Adult female treated with fluorescein acetate under clear field and fluorescence microscope.

16

Figure S1.

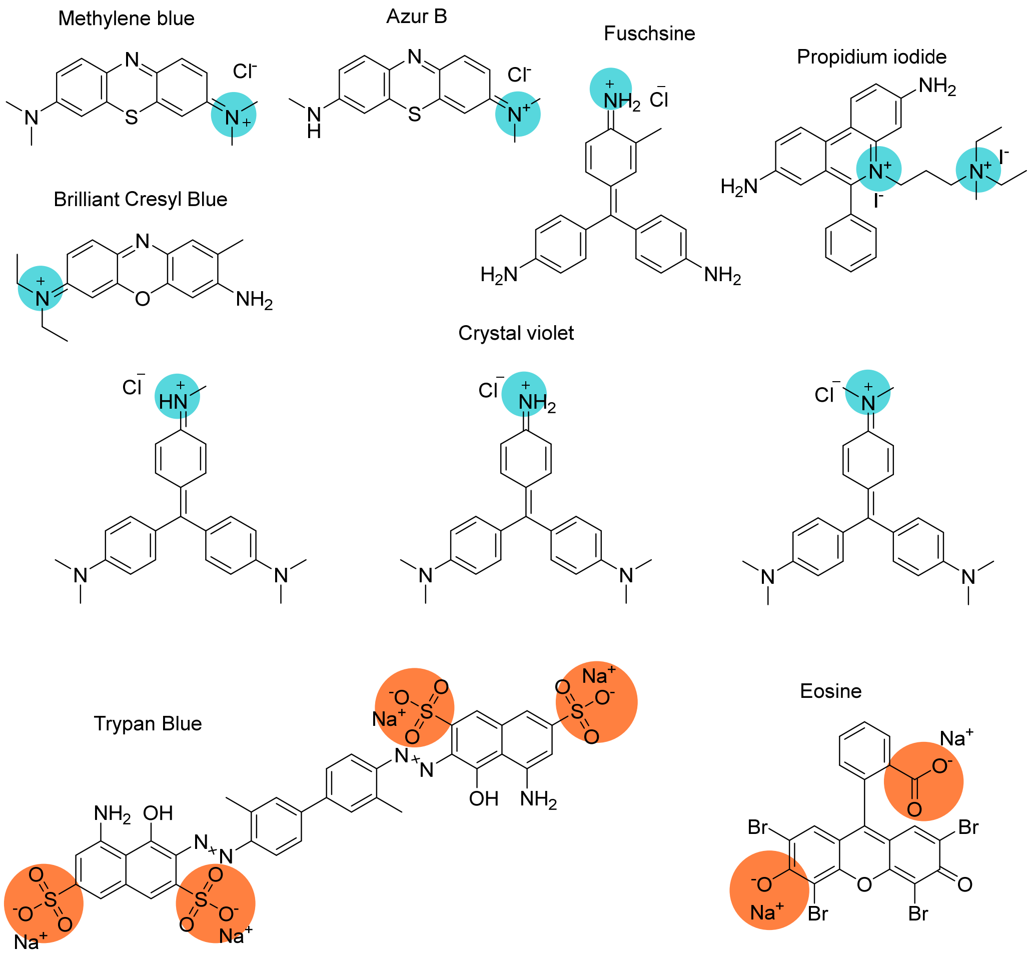
Methylene blue
Azur B
Fuschsine
Propidium iodide
Brilliant Cresyl Blue
Crystal violet
Trypan Blue
Eosine

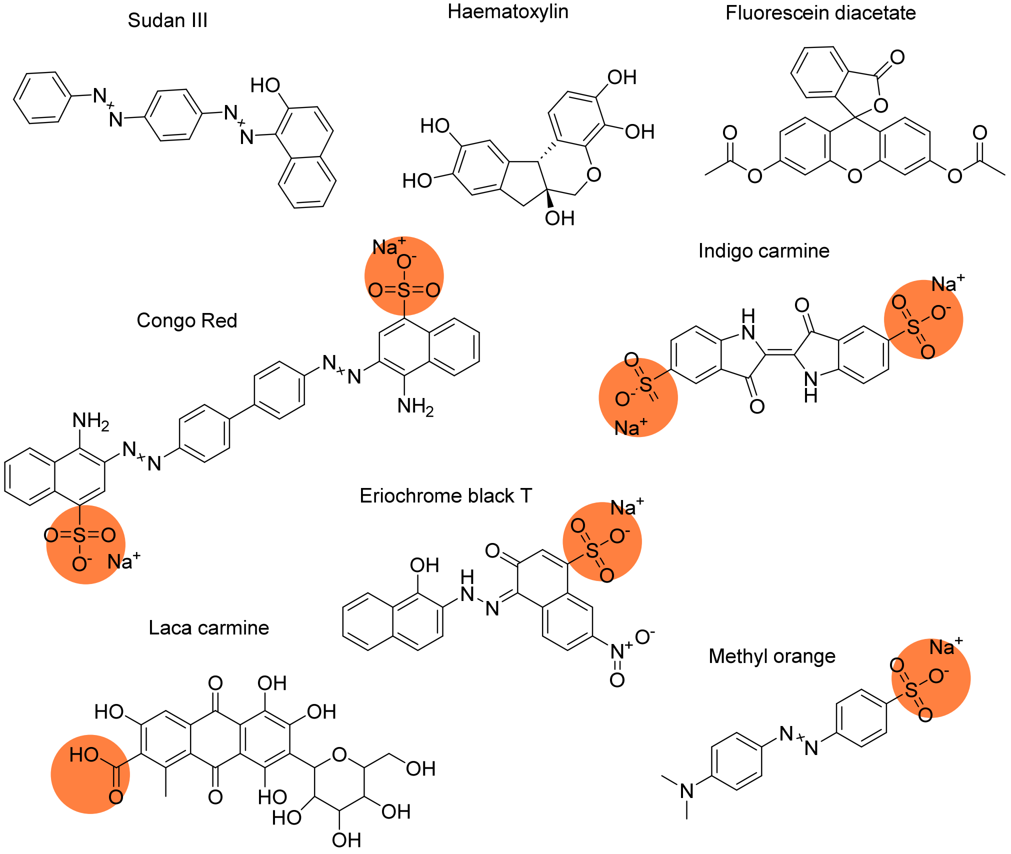
Sudan III
Haematoxylin
Fluorescein diacetate
Congo Red
Indigo carmine
Eriochrome black T
Laca carmine
Methyl orange

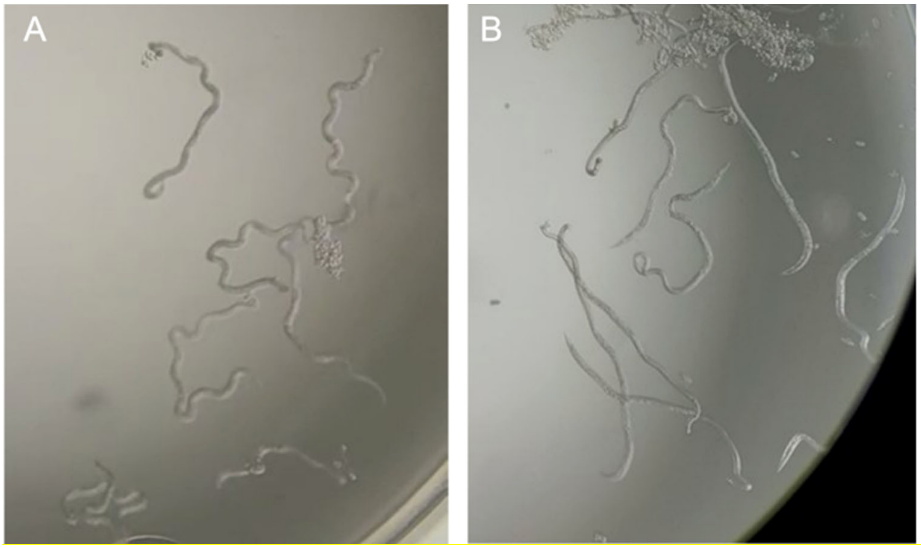

Figure S2: Healthy parthenogenetic adult females of Strongyloides venezuelensis (A) and treated with 10 μM albendazole for 48 hours (B). The serpent shape is lost to a more elongated one. The same shape is observed for adults treated with mebendazole (10 μM) (data not shown).

Table S1

Group		Mean Female	EPGF day 4	EPGF day 5	EPGF day 6	EPGF day 7	EPGF day 8
**Infection**	**G1**	340	0	23141	137471	71954	40190
**Ivermectin**	**G2**	29	160	20534	9559	8544	3290
**MB Gavage**	**G3**	318	0	17652	91673	104635	33039
**MB Water**	**G4**	388	0	19131	80617	74079	26410
**CV Water**	**G5**	182	0	6861	116364	83275	7189

Table S2 Properties calculated with SwissADME

Molecule	Canonical SM	Formula	MW	#Heavy atom	matic heavy a	Fraction Csp3	otatable bon	-bond accept	#H-bond dor	MR	TPSA	iLOGP	XLOGP3	WLOGP	MLOGP
Methylene Blue	CN(c1ccc2c(c	C16H18ClN3S	319,85	21	14	0,25	1	1	0	93,3	47,38	-4,21	3,04	-0,5	2,5
Brilliant Cresyl Blue	CC[N+](=c1cc	C17H20N3O+	282,36	21	14	0,29	2	2	1	86,61	55,06	-0,87	1,86	2,64	1,65
Crystal Violet	C[N+](=C1C=	C25H30ClN3	407,98	29	12	0,24	4	0	0	128,45	9,49	-1,03	0,74	1,46	4,1
Fuschine	Nc1ccc(cc1)C	C20H20ClN3	337,85	24	12	0,05	2	0	3	105,72	77,63	0	4,49	-0,61	3,04
Propidium iodide	CC[N+](CCC[	C27H34I2N4	668,39	33	20	0,3	7	0	2	163,35	55,92	-5,5	6,67	-0,99	0,73
Trypan Blue	Cc1cc(ccc1N=	C34H24N6Na	960,81	62	32	0,06	9	18	4	202,94	404,26	-56,22	4,35	9,64	2,72
Eosine	[O-]C(=O)c1c	C20H6Br4Na	691,85	31	20	0	2	5	0	120,49	93,4	-23,51	5,63	6,12	4,12
Sudan III	Oc1ccc2c(c1N	C22H16N4O	352,39	27	22	0	4	5	1	107,21	69,67	4,02	5,91	7,38	4,15
Haematoxylin	Oc1cc2C[C@	C16H14O6	302,28	22	12	0,25	0	6	5	77,22	110,38	1,69	1,19	1,32	0,49
Fluorescein diacetate	CC(=O)Oc1cc	C24H16O7	416,38	31	18	0,12	4	7	0	107,7	88,13	3,28	3,64	4	3,49
Congo Red	Nc1c(N=Nc2c	C32H22N6Na	696,66	48	32	0	7	10	2	172,95	232,64	-24,06	5,73	9,64	4,12
Indigo Carmine	O=C1/C(=C/2	C16H8N2Na2	466,35	30	12	0	2	8	2	97,34	189,36	-18,14	1,22	1,64	-0,18
Methyl Orange	CN(c1ccc(cc1	C14H14N3Na	327,33	22	12	0,14	4	5	0	79,27	93,54	-9,96	3,33	4,15	2,31
Laca Carmine	OCC1OC(C(C	C22H20O13	492,39	35	12	0,32	3	13	9	112,39	242,51	-1,31	0,53	-1,52	-3,21
Ivermectin a	COC1CC(OC2	C48H74O14	875,09	62	0	0,81	8	14	3	230,77	170,06	5,93	6,34	5,6	1,25
Ivermectin b	COC1CC(OC(	C47H72O14	861,07	61	0	0,81	7	14	3	225,96	170,06	6,62	5,99	5,21	1,09
Edelfosine	CCCCCCCCCC	C27H58NO6P	523,73	35	0	1	27	6	0	146,91	86,86	1,76	7,64	7,56	-0,43
Albendazole	CCCSc1ccc2c	C12H15N3O2	265,33	18	9	0,33	6	3	2	73,22	92,31	1,69	2,81	3,05	2,03
Mebendazole	COC(=O)Nc1	C16H13N3O3	295,29	22	15	0,06	5	4	2	81,75	84,08	1,47	3,01	2,78	1,68
Compound 20	Cc1ccc2c(c1)s	C14H11N3OS	269,32	19	15	0,07	3	3	1	76,6	83,12	1,92	2,84	3,06	1,94
Compound 22	CN1CCN(CC1	C19H21N5OS	367,47	26	15	0,32	4	4	1	112,86	89,6	2,79	3,11	2,05	2,19

Cont

Molecule	Silicos-IT Log	**Consensus L**	ESOL Log S	ESOL Solubil	ESOL Solubil	ESOL Class	Ali Log S	Ali Solubility	Ali Solubility	Ali Class	Silicos-IT Log	Silicos-IT Sol	Silicos-IT Sol	Silicos-IT clas
Methylene Blue	3,24	0,81	-4,17	2,18E-02	6,83E-05	Moderately s	-3,7	6,37E-02	1,99E-04	Soluble	-6	3,21E-04	1,00E-06	Moderately s
Brilliant Cresyl Blue	2,87	1,63	-3,12	2,12E-01	7,52E-04	Soluble	-2,64	6,50E-01	2,30E-03	Soluble	-6,67	6,02E-05	2,13E-07	Poorly solub
Crystal Violet	3,11	1,68	-2,88	5,40E-01	1,32E-03	Soluble	-0,52	1,24E+02	3,03E-01	Very soluble	-6,65	9,09E-05	2,23E-07	Poorly solub
Fuschine	3,41	2,07	-5	3,37E-03	9,97E-06	Moderately s	-5,84	4,88E-04	1,44E-06	Moderately s	-5,58	8,78E-04	2,60E-06	Moderately s
Propidium iodide	1,99	0,58	-8,17	4,49E-06	6,72E-09	Poorly solub	-7,65	1,51E-05	2,25E-08	Poorly solub	-9,5	2,10E-07	3,14E-10	Poorly solub
Trypan Blue	1,28	-7,65	-8,33	4,54E-06	4,73E-09	Poorly solub	-12,55	2,68E-10	2,79E-13	Insoluble	-9,08	7,97E-07	8,30E-10	Poorly solub
Eosine	6,63	-0,2	-8,02	6,58E-06	9,51E-09	Poorly solub	-7,35	3,06E-05	4,42E-08	Poorly solub	-9,64	1,57E-07	2,28E-10	Poorly solub
Sudan III	5,95	5,48	-6,09	2,88E-04	8,18E-07	Poorly solub	-7,15	2,51E-05	7,13E-08	Poorly solub	-8,68	7,29E-07	2,07E-09	Poorly solub
Haematoxylin	1,37	1,21	-2,87	4,10E-01	1,36E-03	Soluble	-3,1	2,38E-01	7,87E-04	Soluble	-2,63	7,10E-01	2,35E-03	Soluble
Fluorescein diacetate	4,29	3,74	-4,88	5,48E-03	1,32E-05	Moderately s	-5,18	2,76E-03	6,62E-06	Moderately s	-7,39	1,68E-05	4,04E-08	Poorly solub
Congo Red	4,29	-0,05	-7,8	1,10E-05	1,58E-08	Poorly solub	-10,38	2,89E-08	4,14E-11	Insoluble	-11,1	5,52E-09	7,93E-12	Insoluble
Indigo Carmine	-0,5	-3,19	-3,66	1,01E-01	2,17E-04	Soluble	-4,79	7,50E-03	1,61E-05	Moderately s	-4,47	1,58E-02	3,38E-05	Moderately s
Methyl Orange	1,63	0,29	-4,11	2,56E-02	7,82E-05	Moderately s	-4,97	3,50E-03	1,07E-05	Moderately s	-4,46	1,14E-02	3,48E-05	Moderately s
Laca Carmine	-0,55	-1,21	-3,28	2,57E-01	5,22E-04	Soluble	-5,19	3,15E-03	6,40E-06	Moderately s	-0,62	1,19E+02	2,42E-01	Soluble
Ivermectin a	2,72	4,37	-8,73	1,62E-06	1,85E-09	Poorly solub	-9,7	1,74E-07	1,99E-10	Poorly solub	-3,89	1,13E-01	1,29E-04	Soluble
Ivermectin b	2,3	4,24	-8,49	2,78E-06	3,23E-09	Poorly solub	-9,34	3,95E-07	4,59E-10	Poorly solub	-3,51	2,67E-01	3,10E-04	Soluble
Edelfosine	4,63	4,23	-6,12	3,99E-04	7,62E-07	Poorly solub	-9,3	2,61E-07	4,97E-10	Poorly solub	-8,62	1,26E-06	2,41E-09	Poorly solub
Albendazole	2,35	2,39	-3,23	1,56E-01	5,90E-04	Soluble	-4,41	1,04E-02	3,93E-05	Moderately s	-4,35	1,19E-02	4,48E-05	Moderately s
Mebendazole	2,55	2,3	-3,74	5,35E-02	1,81E-04	Soluble	-4,44	1,07E-02	3,63E-05	Moderately s	-5,5	9,42E-04	3,19E-06	Moderately s
Compound 20	3,68	2,69	-3,69	5,56E-02	2,06E-04	Soluble	-4,24	1,54E-02	5,70E-05	Moderately s	-5,5	8,46E-04	3,14E-06	Moderately s
Compound 22	3,2	2,67	-4,24	2,11E-02	5,75E-05	Moderately s	-4,66	8,04E-03	2,19E-05	Moderately s	-5,62	8,84E-04	2,40E-06	Moderately s

Cont.

Molecule	log Kp (cm/s)	BB permean	Pgp substrate	P1A2 inhibit	P2C19 inhibit	P2C9 inhibit	P2D6 inhibit	P3A4 inhibit	inski #violati	ose #violatio	ber #violatio	gan #violatio	egge #violati	vailability Sc	AINS #alerts	Brenk #alerts	keness #viol	netic Accessil
Methylene Blue	-6,09	No	Yes	No	No	No	No	No	0	1	0	0	0	0,55	0	2	0	3,44
Brilliant Cresyl Blue	-6,7	Yes	Yes	Yes	No	No	Yes	Yes	0	0	0	0	0	0,55	0	3	0	3,52
Crystal Violet	-8,26	Yes	Yes	No	No	No	No	No	0	0	0	0	0	0,55	1	2	1	4,11
Fuschine	-5,17	No	No	Yes	No	No	Yes	No	0	1	0	0	0	0,55	0	2	1	3,47
Propidium iodide	-5,64	No	Yes	Yes	No	No	No	No	1	3	0	0	2	0,55	0	5	2	3,39
Trypan Blue	-9,07	No	Yes	No	No	No	No	No	2	4	1	2	3	0,17	1	3	3	5,19
Eosine	-6,52	No	Yes	No	No	No	No	No	1	2	0	1	2	0,55	0	1	2	3,55
Sudan III	-4,25	No	Yes	No	Yes	No	No	No	0	1	0	1	1	0,55	1	1	2	3,04
Haematoxylin	-7,3	No	Yes	Yes	No	No	No	No	0	0	0	0	0	0,55	1	1	0	3,74
Fluorescein diacetate	-6,26	No	No	Yes	Yes	Yes	No	Yes	0	0	0	0	0	0,55	0	1	2	3,81
Congo Red	-6,48	No	Yes	No	No	No	No	No	2	3	1	2	3	0,17	1	3	2	4,22
Indigo Carmine	-8,28	No	Yes	No	No	No	No	No	0	0	1	1	1	0,55	1	2	1	3,02
Methyl Orange	-5,93	No	Yes	No	No	No	No	No	0	0	0	0	0	0,55	2	2	0	2,95
Laca Carmine	-8,93	No	No	No	No	No	No	No	2	2	1	1	3	0,11	2	2	1	5,04
Ivermectin a	-7,14	No	Yes	No	No	No	No	No	2	4	1	1	4	0,17	0	1	3	10
Ivermectin b	-7,3	No	Yes	No	No	No	No	No	2	3	1	1	4	0,17	0	1	2	10
Edelfosine	-4,07	No	Yes	No	No	No	No	Yes	1	4	1	1	2	0,55	0	2	3	6,43
Albendazole	-5,92	No	No	Yes	No	No	No	No	0	0	0	0	0	0,55	0	0	0	2,41
Mebendazole	-5,96	No	No	Yes	No	No	No	No	0	0	0	0	0	0,55	0	0	0	2,26
Compound 20	-5,93	No	No	Yes	Yes	Yes	No	Yes	0	0	0	0	0	0,55	0	0	0	2,43
Compound 22	-6,33	No	Yes	Yes	No	Yes	Yes	Yes	0	0	0	0	0	0,55	0	0	1	3,09

Table S3.

name	L3	Ad	formula	molwt	rb	glob	pbf	func_group	Charge
Methylene Blue	+	+	C16H18ClN3S	320	1	0,01	0,27	No Amine	1
Brilliant Cresyl Blue	+	+	C17H20N3O+	282	2	0,03	0,56	No Amine	1
Crystal Violet	+		C25H30ClN3	408	4	0,12	0,94	No Amine	1
Fuchsine	+		C20H20ClN3	338	2	0,13	0,73	No Amine	1
Propidium iodide	+		C27H34I2N4	668	7	0,11	0,95	No Amine	2
Trypan Blue	+		err	err	err	err	err	err	1
Eosine			C20H6Br4Na2O5	692	2	0,18	0,66	No Amine	-4
Sudan III			C22H16N4O	352	4	0,13	1,17	No Amine	-2
Haematoxylin			C16H14O6	302	0	0,17	0,98	No Amine	0
Fluorescein diacetate			C24H16O7	416	4	0,12	1,03	No Amine	0
Congo Red			C32H22N6Na2O6S2	697	7	0,01	0,67	No Amine	-2
Indigo Carmine			C16H8N2Na2O8S2	466	2	0,01	0,20	No Amine	-1
Methyl Orange			C14H14N3NaO3S	327	4	0,03	0,63	No Amine	-1
Carmine			C22H20O13	492	3	0,05	0,70	No Amine	-1
Ivermectin a			C48H74O14	875	8	0,11	1,54	No Amine	0
Ivermectin b			C47H72O14	861	7	0,08	1,40	No Amine	0
Edelfosine			C27H58NO6P	524	27	0,03	1,06	No Amine	0
Albendazole			C12H15N3O2S	265	5	0,07	0,74	No Amine	0
Mebendazole			C16H13N3O3	295	4	0,03	0,65	No Amine	0
Compound **20**			C14H11N3OS	269	2	0,01	0,35	No Amine	0
Compound **22**	+	+	C19H21N5OS	367	3	0,01	0,47	Tertiary Amine	1

Table S4. Activities against different population densities (1-3 adults, 10-15 adults and 50-70 adults) at 72 hours with 10 μM treatments. Culture control was 1% DMSO as basal. Healthy: Normal and vivid movement and shape, no differences against control; Slightly affected: slower movement or shape different from the control; Affected: abnormal movements and shape clearly different from the control and clearly incompatible with normal viability, no deaths; Dead: immobile larvae. Increase in potencies can be observed when decreasing the number of adults per well

**Number**	**Compound**	Activity against adults 10µM 72h **1-3 adults**	Activity against adults 10µM 72h **10-15 adults**	Activity against adults 10µM 72h **50-70 adults**
**1**	Methylene blue	**Dead**	**Dead**	**Dead**
**2**	Brilliant cresyl blue	**Dead**	**Affected / Some deaths**	**Affected**
**3**	Crystal violet	Slightly affected	Slightly affected	Slightly affected
**4**	Fuchsine	Healthy	Healthy	Healthy
**5**	Propidium iodide	**Affected / Some deaths**	**Affected / Some deaths**	**Affected / Some deaths**
**16**	Ivermectin	**Affected**	**Affected**	Slightly affected
**18**	Albendazole	**Affected**	Slightly affected	Slightly affected
**19**	Mebendazole	**Affected**	Slightly affected	Slightly affected
**20**	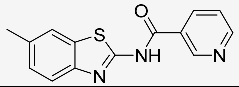	Healthy	Healthy	Healthy
**22**	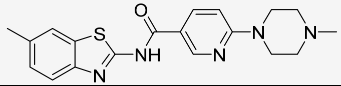	**Affected**	**Affected**	Slightly affected

Video S1

Video 1S.mp4
